# Chemogenetic Silencing of Differentiating Cortical Neurons Impairs Dendritic and Axonal Growth

**DOI:** 10.3389/fncel.2022.941620

**Published:** 2022-07-15

**Authors:** Ina Gasterstädt, Max Schröder, Lukas Cronin, Julian Kusch, Lisa-Marie Rennau, Brix Mücher, Stefan Herlitze, Alexander Jack, Petra Wahle

**Affiliations:** ^1^Developmental Neurobiology, Faculty of Biology and Biotechnology, Ruhr University Bochum, Bochum, Germany; ^2^Department of General Zoology and Neurobiology, Faculty of Biology and Biotechnology, Ruhr University Bochum, Bochum, Germany

**Keywords:** postnatal development, dendritogenesis, neurite growth, pyramidal cell, metabotropic, hM4Di

## Abstract

Electrical activity is considered a key driver for the neurochemical and morphological maturation of neurons and the formation of neuronal networks. Designer receptors exclusively activated by designer drugs (DREADDs) are tools for controlling neuronal activity at the single cell level by triggering specific G protein signaling. Our objective was to investigate if prolonged silencing of differentiating cortical neurons can influence dendritic and axonal maturation. The DREADD hM4Di couples to G_i/o_ signaling and evokes hyperpolarization *via* GIRK channels. HM4Di was biolistically transfected into neurons in organotypic slice cultures of rat visual cortex, and activated by clozapine-N-oxide (CNO) dissolved in H_2_O; controls expressed hM4Di, but were mock-stimulated with H_2_O. Neurons were analyzed after treatment for two postnatal time periods, DIV 5-10 and 10-20. We found that CNO treatment delays the maturation of apical dendrites of L2/3 pyramidal cells. Further, the number of collaterals arising from the main axon was significantly lower, as was the number of bouton terminaux along pyramidal cell and basket cell axons. The dendritic maturation of L5/6 pyramidal cells and of multipolar interneurons (basket cells and bitufted cells) was not altered by CNO treatment. Returning CNO-treated cultures to CNO-free medium for 7 days was sufficient to recover dendritic and axonal complexity. Our findings add to the view that activity is a key driver in particular of postnatal L2/3 pyramidal cell maturation. Our results further suggest that inhibitory G protein signaling may represent a factor balancing the strong driving force of neurotrophic factors, electrical activity and calcium signaling.

## Introduction

During neocortical development, electrical activity is a major factor for the maturation of the nervous system including neurochemical and structural differentiation of neurons and the formation of functional networks. In the first week after birth, early activity is triggered by cholinergic input *via* muscarinic receptors on subplate neurons, which subsequently elicit glutamatergic network oscillations assumed to promote dendritic and axonal maturation and synapse formation (Ghosh et al., 1990; Kilb and Luhmann, 2003; Dupont et al., 2006; Yang et al., 2016; Molnár et al., 2020). Glutamate receptor signaling is important for dendritic growth (Wong and Ghosh, 2002). For pyramidal neurons, AMPA receptor isoforms with prolonged channel open time elicit apical dendritic growth and branching under the contribution of voltage-gated calcium channels and NMDA receptors (Hamad et al., 2011). Also, type I transmembrane AMPA receptor regulatory proteins promote the dendritogenesis in immature pyramidal cells by enhanced trafficking of endogenous AMPA receptors (Hamad et al., 2014). GluN2B-containing NMDA receptors by contrast regulate differentiation of pyramidal cell basal dendrites (Wedzony et al., 2005; Gonda et al., 2020). Further, overexpressing the kainate receptor GluK2 increases dendritic complexity of pyramidal cell apical dendrites of cortical layers II/III (L2/3) (Jack et al., 2018). Kainate at low concentrations elicits network activity (Lauri et al., 2005; Valbuena and Lerma, 2016) which leads to dendritic growth of L2/3 pyramidal neurons (Jack et al., 2018). Moreover, it triggers for instance axonal growth of hippocampal neurons (Tashiro et al., 2003) whereas a blockade of AMPA receptors suppresses axonal branching of supragranular neurons (Uesaka et al., 2005). The underlying mechanism triggered by low kainate dosage is thought to involve metabotropic signaling within interneuronal terminals, thus downregulating GABA release in a Gi/o-sensitive manner, presumably *via* GABA_B_ or endocannabinoid receptors (for review see Valbuena and Lerma, 2016).

Early after birth, L2/3 pyramidal neurons require GABA_A_ receptor-mediated depolarization (Cancedda et al., 2007) as well as GABA_B_ receptor-mediated cAMP signaling (Bony et al., 2013). The latter is non-hyperpolarizing in perinatal rodent because GABA_B_ receptors, while already highly expressed, are not yet coupled to GIRK channels (López-Bendito et al., 2002; Bony et al., 2013). Further, GABA_B_ receptor activation can impair axonal elongation (Bird and Owen, 1998; Ferguson and McFarlane, 2002; Xiang et al., 2002). Similarly, endocannabinoid CB1 receptors are highly expressed on developing corticofugal axons influencing growth cone collapse and proper targeting (Roland et al., 2014) and mediate formation and stabilization of GABAergic axonal boutons (Liang et al., 2021). Loss of CB1 receptor signaling evokes aberrant apical dendritic sprouts of migrating pyramidal neurons of the cortical plate, which impairs their migration (Díaz-Alonso et al., 2017). The cannabinoid action depends on the type of G protein and the spatiotemporal context. Together, extensive knowledge exists on the role of ionotropic signaling and depolarization in a neurodevelopmental context. Yet, with the exception of neurotrophic signaling (Valnegri et al., 2015, for review), much less is known on metabotropic signaling. Moreover, compared to growth-promoting actions, growth-inhibiting mechanisms are less often studied. Activation of G_i/o_ signaling leads to GIRK channel-mediated hyperpolarization of cultured hippocampal neurons (Armbruster et al., 2007). Here, we report that the differentiation of individual pyramidal neurons in an otherwise electrically active organotypic network can be influenced by prolonged silencing *via* the G_i/o_ coupled DREADD hM4Di.

## Materials and Methods

### Preparation of Organotypic Cultures

We investigated organotypic slice cultures of rat visual cortex at two postnatal time points, DIV 10 and DIV 20, with quantitative assessment of dendritic and axonal maturation of pyramidal cells and multipolar interneurons. Cultures were prepared from pigmented Long-Evans rats at P1 as described (Gasterstädt et al., 2020; Gonda et al., 2020). The visual cortex was extracted and cut sagittally into 350 μm thick slices (McIlwain tissue chopper, Ted Pella, Redding, CA, United States). Slices were mounted on coverslips with a coagulate of plasma (2:1 chicken/bovine) and thrombin and cultured at 37°C in roller-tubes with 700 μl semi-artificial medium containing: 10% adult horse serum, 25% Hank’s balanced Salt Solution, 50% Eagle’s Basal Medium (Pan-Biotech, Aidenbach, Germany), 0.5% NeuroCult™ SM1 Neuronal Supplement (STEMCELL Technologies, Cologne, Germany, Cat. #05711), 1 mM L-Glutamine (GIBCO, Karlsruhe, Germany), and 0.65% D-Glucose (Merck, Darmstadt, Germany). Enhanced glial differentiation was prevented at DIV 2 with a mixture of uridine, cytosine-ß-D-arabinofuranoside and 5-fluoro-2′-deoxyuridin (all from Sigma-Aldrich, Deisenhofen, Germany) for 24 h. Slices from every individual animal (4–5 animals per batch) were allocated to all experimental conditions run with this batch of cultures.

### Plasmids Transfection

All plasmids (Table 1) were prepared as endotoxin-free solutions using the EndoFree Plasmid Maxi Kit (Qiagen, Hilden, Germany, Cat. #12362). Plasmid stocks were diluted to 1 μg/μl and stored at −20°C. Cartridges were prepared by coating 7 mg gold microparticles (1 μm diameter; MaTeck GmbH, Jülich, Germany) with 10 μg plasmid encoding EGFP and/or GCaMP6m and/or 15 μg plasmid encoding hM4Di (Table 1). Biolistic transfection (Helios Gene Gun, Bio-Rad Laboratories, Feldkirchen, Germany) was done at DIV 4 as described with 180 psi helium pressure (Gasterstädt et al., 2020). The mCherry-tagged hM4Di was functional in the calcium imaging, but was not sufficient to label the transfectants completely; in addition, receptors with large tags may not always become trafficked to the proper cellular localizations. Therefore, HA-tagged hM4Di was used to analyze the hM4Di protein in the cells with immunohistochemistry. HA-tagged hM4Di was also used for morphometry, but since it was not able to fully label the cells, EGFP was co-expressed to yield complete cytosolic labeling of dendrites and axons, and was used to stain for morphometry. Coexpression rates are high with biolistic transfection (Wirth et al., 2003; Hamad et al., 2011).

**TABLE 1:** Plasmids and reagents.

Plasmid	Promoter	Source, option	Catalog number
pEGFP-N1	CMV	Clontech, Heidelberg, Germany; used to completely label the neurons	cat# 632370
pAAV-CW3SL-EGFP	CMV	Choi et al. (2014) (gift from Bong-Kiun Kaang); for HEK cell labeling	RRID:Addgene_61463
pGP-CMV-GCaMP6m	CMV	Chen et al. (2013) (gift from Douglas Kim); for calcium imaging	RRID:Addgene_40754
pAAV-hSyn-hM4D(Gi)-mCherry	Human synapsin 1	Armbruster et al. (2007) (gift from Stefan Herlitze); for calcium imaging	RRID:Addgene_50475
pcDNA5/FRT-HA-hM4D(Gi)	CMV	Armbruster et al. (2007) (gift from Stefan Herlitze); for morphometry	RRID:Addgene_45548

**Antibody**	**Dilution**	**Source**	**Catalog number**

Mouse anti-GFP	1:1000	Clone GSN24, Sigma-Aldrich, Deisenhofen, Germany	RRID:AB_563117
Biotinylated goat anti-mouse	1:1000	Agilent, Ratingen, Germany	RRID:AB_2687905
ABC reagent	As recommended	Vector Laboratories Inc., Burlingame, CA, United States	RRID:AB_2336827
Donkey anti-mouse Alexa-568	1:1000	Thermo Fisher Scientific, Waltham, MA, United States	RRID:AB_2534013
Mouse anti-HA.11	1:1000	BioLegend, San Diego, CA, United States	RRID:AB_2565335
Mouse anti-mCherry	1:1000	Takara Bio, Saint-Germain-en-Laye, France	RRID:AB_2307319

### Pharmacological Treatment

Designer receptors exclusively activated by designer drugs (DREADD) are genetically modified G protein-coupled receptors (GPCRs). The G_i/o_-coupled hM4Di DREADD was generated by introducing two point mutations (Y149C^3.33^/A239G^5.46^) in strictly conserved areas of the human muscarinic acetylcholine receptor ligand binding site (Armbruster et al., 2007). These modified receptors are characterized by a decreased affinity for their natural agonist acetylcholine (and also carbachol) and a high affinity for the synthetic ligand CNO (Armbruster et al., 2007; Bonaventura et al., 2019). DREADDs have proven to be ideal tools for behavioral studies with acute stimulation. However, DREADDs have rarely been used during development and with prolonged stimulation over several days.

We assessed two postnatal time points. For DIV 10, cultures were stimulated once daily between DIV 5 to DIV 10 with 3 μM CNO (dissolved in ddH_2_O; ENZO Life Sciences, Lausen, Germany). Medium was changed every second day. Control cultures were mock-stimulated with ddH_2_O. For DIV 20, cultures were treated once daily from DIV 10 to DIV 20. For the recovery, cultures were stimulated with CNO from DIV 5 to DIV 12. At DIV 13, a medium change removed the remaining CNO, and cultures recovered in normal medium until DIV 20.

### Immunostaining

Cultures were fixed with 37°C 4% paraformaldehyde in 0.1 M phosphate buffer pH 7.4 for 30 min, rinsed, permeabilized with Triton X-100 in phosphate buffer for 30 min, blocked with 5% BSA in TBS, and incubated in mouse anti-GFP antibody for 12–24 h, followed by biotinylated goat anti-mouse for 2 h, followed by ABC reagent for 2 h, and a H_2_O_2_ induced HRP reaction with 3,3′-diaminobenzidine (DAB; Sigma-Aldrich, Deisenhofen, Germany). The DAB product was intensified for 30 s with 1% OsO_4_ (Sigma-Aldrich, Deisenhofen, Germany). Cultures were dehydrated and coverslipped with DEPEX (Sigma-Aldrich, Deisenhofen, Germany). For detection of hM4Di expression, a sequence containing a HA-tag was transfected, detected with mouse anti-HA.11 epitope tag antibody overnight, and visualized with secondary antibody donkey anti-mouse Alexa Fluor-568.

### Confocal Calcium Imaging

Neurons transfected with hM4Di-mCherry and GCaMP6m were recorded between DIV 15 and 20 to check the effect of an inhibitory DREADD in postnatal neurons. Briefly, the cultures were rinsed several times with oxygenated HEPES-ACSF, and allowed to adjust to conditions for 1 h in the roller incubator. The mCherry served to detect transfectants and to determine the cell type. Baseline activity was recorded for 5–10 min. CNO in HEPES-ACSF was superfused at a final concentration of 3 μM. Activity of transfectants was recorded with a Leica TCS SP5 confocal microscope (Leica, Mannheim, Germany) with a × 10 objective at 1,400 Hz and 2.7 frames/s as described (Hamad et al., 2014; Jack et al., 2018).

### Electrophysiology in HEK Cells

For the *in vitro* verification of HA-hM4Di-mediated currents, HEK GIRK 1/2 cells (HEK293 cells stably expressing GIRK1 and GIRK2 subunits, kindly provided by Dr. Tinkler UCL London, GB) were maintained in Dulbecco’s modified Eagle’s medium – high glucose (Sigma-Aldrich, Deisenhofen, Germany), supplemented with 10% fetal bovine serum (GIBCO), 1% penicillin/streptomycin (GIBCO, Karlsruhe, Germany) and 0.5 mg/ml Geneticin (GIBCO, Karlsruhe, Germany) at 37°C in a humidified incubator at 5% CO_2_. The cells were seeded on 35 × 10 mm cell culture dishes (CELLSTAR^®^, Greiner Bio-One GmbH, Frickenhausen, Germany) and co-transfected with CW3SL-EGFP and HA-tagged hM4Di (same clone as used for the morphometry) using FuGENE^®^ HD (Promega, Walldorf, Germany) according to the manufacturer’s protocol 12–24 h before recordings.

After the visualization of co-transfected cells by EGFP emission (excitation with 471 nm, Polychrome V, Till Photonics) at an inverted microscope (Axiovert, Zeiss, Oberkochen, Germany) and exchanging the growth medium for an external solution (20 mM NaCl, 120 mM KCl, 2 mM CaCl^2^, 1 mM MgCl^2^, 10 mM HEPES, to pH7.3 with KOH) whole-cell patch clamp recordings of DREADD-mediated GIRK currents were obtained with patch pipettes (2–4 MΩs, MPC325, Sutter instrument, Novato, CA, United States) filled with an internal patch solution (100 mM L-aspartic acid potassium, 40 mM KCl, 40 mM MgATP, 10 mM HEPES, 5 mM NaCl, 2 mM EGTA, 2 mM MgCl^2^, 0.01 mM GTP, to pH7.3 with KOH). Signals were forwarded through an USB amplifier (EPC10, HEKA, Reutlingen, Germany), digitized and filtered with a 10-kHz 3-pole Bessel filter in series with a 2.9-kHz 4-pole Bessel filter and monitored by PatchMaster (v2 × 52, HEKA, Reutlingen, Germany), which also served to control voltage, polychrome and the pneumatic application system (PV820, World Precision Instruments, Friedberg, Germany) for the application of 10 μM CNO or vehicle. HEK293 cells were voltage-clamped at −60 mV and their baseline recorded for 8 s. Subsequent application of CNO or vehicle directly to the recorded cell lasted for 2 s. Afterward the cells were monitored for an additional 10 s period. The size and presence of GIRK currents was assessed with IGOR PRO 6.11 (WaveMetrics, Portland, OR, United States). The electrical current density per capacitance elicited by CNO or vehicle were then compared in SigmaPlot 12.5 (Systat, Chicago, United States) and visually processed with the help of CorelDRAWX6 (Corel Corporation, Ottawa, Canada).

### Morphometry

Neurons immunostained for the co-expressed EGFP were reconstructed at DIV 10 and DIV 20 with the Neurolucida system (MicroBrightField, Inc., Williston, VT, United States) by trained observers blinded to conditions. A second observer, also blinded to conditions, crosschecked all reconstructions for correctness and classification. Pyramidal cells and multipolar interneurons were classified by established criteria of dendritic and axonal patterns (Hamad et al., 2011; Jack et al., 2018; Gasterstädt et al., 2020). Pyramidal neurons of layers II/III (L2/3) have an apical dendrite reaching into layer I, and those of layers V/VI (L5/6) have an apical dendrite ending in middle layers. Thick and thin tufted large L5 pyramidal cells were too rarely transfected and were excluded. For analysis of pyramidal cell axons, the first 500 μm of the descending main axon were reconstructed and the collaterals and bouton terminaux were counted, and reported normalized to 100 μm. Interneurons were divided into basket cells and non-basket cells. The group of basket cells may also contain chandelier cells, which have a delayed maturation (Pan-Vazquez et al., 2020) and can not be safely assessed during the time windows. Basket neurons have highly branched axons with irregular-sized boutons forming dense local or horizontal plexus. The non-basket group comprised bitufted, arcade and Martinotti neurons with vertical-columnar projections, which can be clearly distinguished from basket cells also by their more delicate bouton morphology. For bouton analysis of basket cells at DIV 20, 100x photomicrographs of boutons were analyzed as reported (Engelhardt et al., 2018) with ImageJ to determine bouton size [in μm^2^]; assessment was done by observers who were blinded to condition.

### Statistical Analysis

Bar and box plots, Sholl plots, and statistical analyses were done with Sigma Plot 12.3 (Systat, Chicago, IL, United States). For calcium imaging data, non-parametric ANOVA on ranks tests with corrections for multiple testing when appropriate (Bonferroni’s or Dunn’s test) were conducted. We discovered rather mild effects at DIV 10 and at DIV 20 selectively in L2/3 apical dendrites. Neurons from >7 culture batches went into analysis. To eliminate interbatch variability we normalized each batch (each individual experiment) to the average of its control neurons which was set to 1. To rule out false positives, we present the original values in a Table. We tested with a Mann-Whitney rank sum test the H_2_O mock-stimulation vs. CNO treatment. The number of independent preparations and neurons analyzed is given in the graphs, tables, or legends.

## Results

### Cortical Neurons Display HM4Di Protein Expression

**FIGURE 1:**
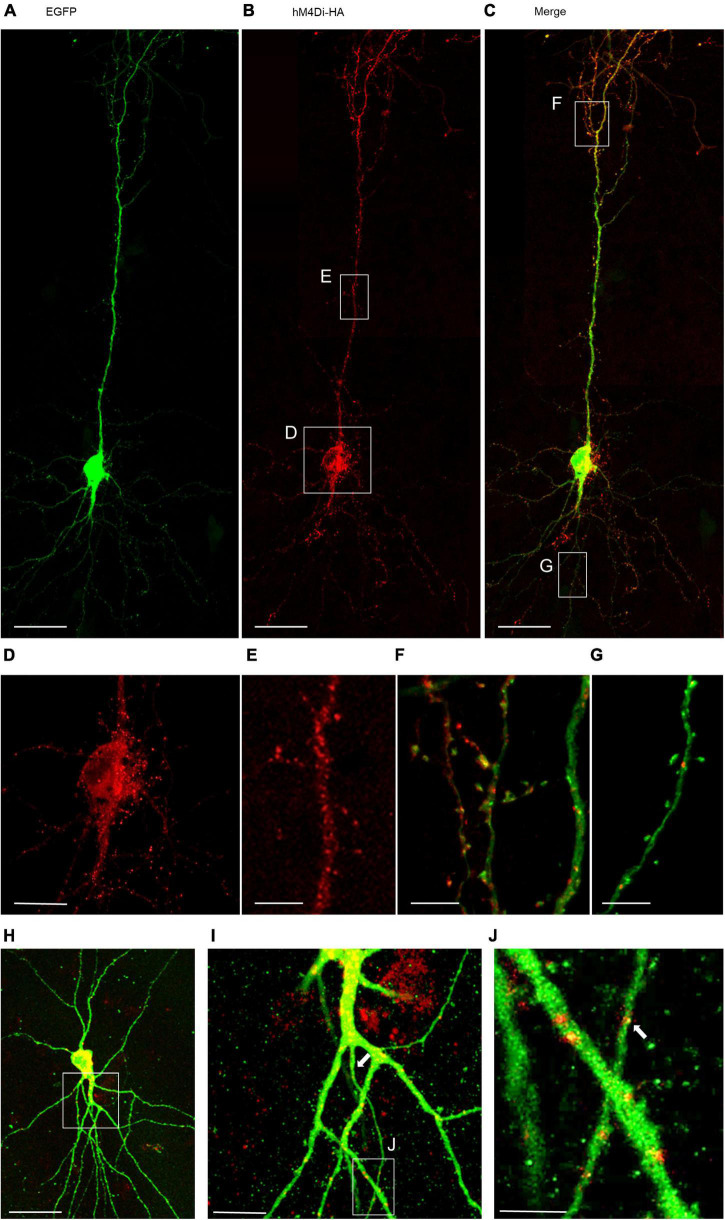
Immunofluorescent staining of a pyramidal neuron expressing HA-tagged hM4Di. Co-transfection of plasmids encoding EGFP **(A)** and HA-tagged hM4Di visualized with mouse anti-HA/Alexa-568 **(B)**, and merged **(C,H)**. The hM4Di receptor was expressed heterogeneously throughout the cell; with a high density in the soma **(D)**, the trunk of the apical dendrite **(E,I)** and in the apical tuft **(F)**. HM4Di clusters are located at the dendritic membrane sometimes associated with spines **(F)**. Isolated clusters of hM4Di were present in basal dendrites **(G)** and in the axon **(J)**. Pial surface is to the top. White arrows indicate the axon. Scale: 100 μm for panels **(A–C,H)**; 35 μm **(D,I)**; 25 μm **(E–G)**; 10 μm **(J)**.

The natural G_i_/o-linked muscarinic receptors M_2_ and M_4_ have been reported in pyramidal cells and multipolar interneurons (Hájos et al., 1998; Mrzljak et al., 1998; Zheng et al., 2011). Biolistic overexpression of receptors in slice-cultured cortical neurons may work well (Hamad et al., 2011; Jack et al., 2018), but may also fail and not produce the desired protein (Gonda et al., 2020) To confirm that biolistic overexpression yields hM4Di protein which, moreover, remains in the neurons for several days we transfected EGFP at DIV 4 to visualize the neurons and a HA-tagged hM4Di variant to ensure unobstructed receptor trafficking. Imaging at DIV 20 (Figures 1A–C,H) revealed HA-hM4Di immunopositive clusters and puncta in somata and dendrites (Figures 1D–G,I,J). Somata showed a high density of hM4Di puncta overlapping with cytosolic EGFP (Figures 1C,H). Apical dendrites had more hM4Di puncta and clusters than basal dendrites, and the puncta appeared close to the membrane and sometimes associated with spines (Figures 1E–G). Small clusters of HA-hM4Di protein could be detected in axons of pyramidal cells (Figures 1H–J) in line with the known presynaptic localization of G_i/o_-coupled receptors. Expression strength of HA-hM4Di was variable among pyramidal cells (Figures 1C,H). Together, this suggested that cortical neurons are capable of expressing and trafficking hM4Di protein into the plasma membrane.

### HM4Di Is Functional in Cortical Neurons

**FIGURE 2:**
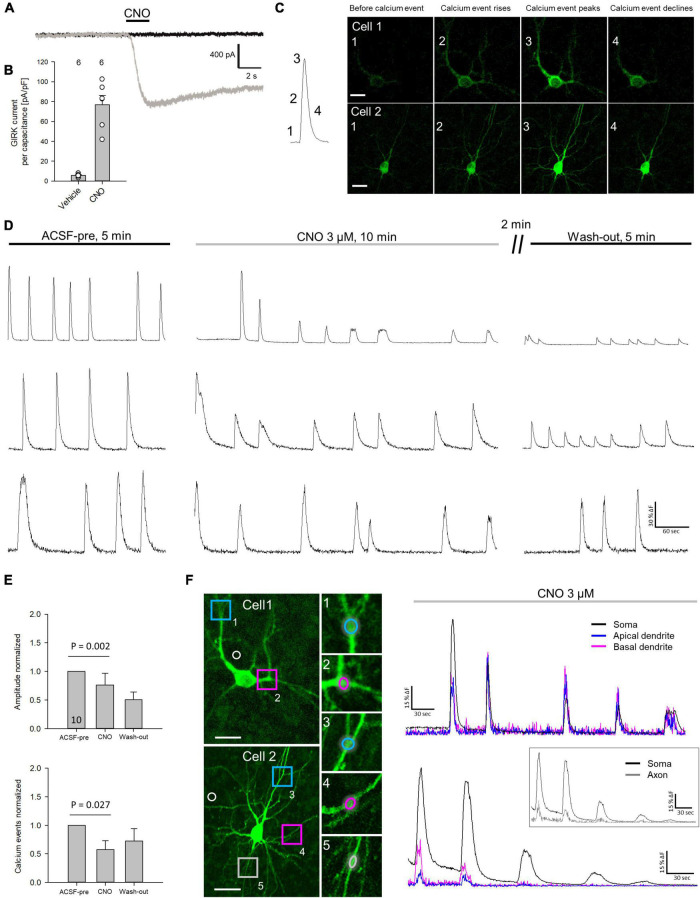
Activation of hM4Di reduces neuronal activity. **(A)** Representative recording of a HA-hM4Di-transfected HEK cell after CNO application. **(B)** Measurement of GIRK channel-mediated currents after hM4Di activation normalized to cell volume. Number of analyzed cells is given above the bars. **(C)** Representative calcium event. The numbers indicate baseline activity (1), and the rise (2), peak (3), and decline (4) of a spontaneous calcium event. Representative images of calcium events of two mCherry-hM4Di/GCaMP6m transfected neurons (cells 1, 2). **(D)** Representative traces of spontaneous calcium events of three selected neurons recorded between DIV 15 and 20. Spontaneous calcium events were measured for 5 min in ACSF, followed by 10 min recording under 3 μM CNO, and 5 min after wash-out. **(E)** Quantitative analysis (mean ± s.e.m.) of somatically recorded calcium event amplitude and frequency in ACSF under CNO and after wash-out. Number of analyzed neurons (DIV 10–20) are given in the bar. **(F)** Analysis of calcium events under CNO application in soma, apical and basal dendrites of cell 1, and soma, dendrites and axon of cell 2. ROIs are indicated; white circles indicate ROIs used for subtraction of background fluorescence. Statistics: Anova on ranks, ACSF-pre vs. CNO. Scale bars: 25 μm in panel **(C)**, and cell overviews in panel **(F)**.

The functionality of the HA-tagged hM4Di construct was first tested in HEK293 cells expressing the GIRK1/2 subunits. Application of 10 μM CNO led to receptor-induced GIRK currents with 76.7 ± 9.4 pA/pF, which is comparable to experimental results described previously (Armbruster et al., 2007; Figures 2A,B). To confirm functional expression in cortical neurons calcium imaging was performed using co-transfection of mCherry-tagged hM4Di and GCaMP6m (Figure 2C). Only neurons spontaneously active during the recording period were considered, and traces of three neurons are shown (Figure 2D). First, baseline activity was monitored for 5 min. To activate hM4Di 3 μM CNO was washed in, and the cells were recorded for 10 min. After wash-out for about 2 min, the cells were recorded for another 5 min. The frequency and the amplitude of calcium events were significantly reduced after activation of hM4Di, and the depression of activity outlasted the CNO application in all cells recorded (Figures 2D,E). Since the distribution of hM4Di was heterogeneous within the neurons, the calcium signal was analyzed in soma, dendrites and axon (Figure 2F). After CNO wash-in, calcium signals declined in soma and dendrites. The calcium signal in the axon seemed to remain at baseline after inhibition of the soma as expected with a silencing of the cell. Together, the experiments suggested that hM4Di is a useful tool to reduce the activity of individual transfected cortical neurons.

### Silencing *via* HM4Di Impairs Pyramidal Cell Apical Dendritic Complexity

**FIGURE 3:**
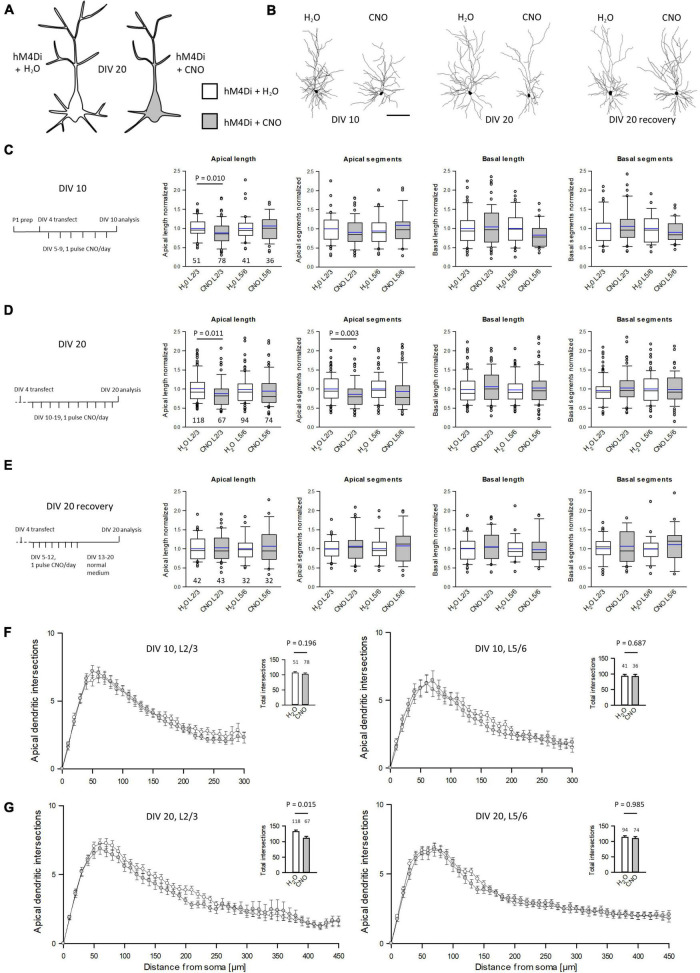
Prolonged reduction of activity *via* hM4Di reduces apical complexity of supraganular pyramidal cells. **(A)** Prolonged activation of hM4Di was hypothesized to reduce dendritic complexity. **(B)** Skeletal drawings of control (left) and CNO-stimulated (right) L2/3 pyramidal cells at DIV 10, DIV 20 and DIV 20 recovery. **(C)** Stimulation protocol and morphometric analysis of L2/3 and L5/6 pyramidal cells at DIV 10. **(D)** Stimulation protocol and morphometric analysis of L2/3 and L5/6 pyramidal cells at DIV 20. **(E)** Stimulation protocol and morphometric analysis of L2/3 and L5/6 pyramidal cells at DIV 20 after recovery. Values were normalized to the average of batch-internal control cells. Black lines in the box plots represent the median and blue lines indicate the mean. **(F)** Sholl analysis and total intersections of L2/3 and L5/6 pyramidal cell apical dendrites at DIV 10. **(G)** Sholl analysis and total intersections of L2/3 and L5/6 pyramidal cell apical dendrites at DIV 20. Numbers of analyzed neurons are given below the box plots or above the bars. Statistics: Mann–Whitney rank sum test H_2_O control vs. CNO, *P*-values are reported. Scale bar in panel **(B)** 100 μm.

Our working hypothesis was that silenced neurons remain hypomorphic (Figure 3A). To test this, neurons were transfected with hM4Di and EGFP at DIV 4 and treated daily with CNO, or water as control, from DIV 5–10 or DIV 10–20. After staining for EGFP, pyramidal cells were reconstructed (Figure 3B). To compensate for interbatch variability, values of length and segment number were normalized to the average of each batch-internal control cell population. Raw data and *P*-values are shown in Supplementary Table 1. At DIV 10, hM4Di activation resulted in a significant delay of apical dendritic length of L2/3 pyramidal cells (Figure 3C). Silencing from DIV 10–20 (Figure 3D) led to an even stronger effect in that length and branching of L2/3 pyramidal cell apical dendrites were reduced. A recovery experiment was performed to analyze if the delay in apical dendritic maturation is reversible. Indeed, after withdrawal of CNO apical dendritic complexity of L2/3 pyramidal cells normalized to that of control cells (Figure 3E). Sholl analyses were performed to analyze dendritic complexity. No difference was found at DIV 10, confirming an effect on apical length, but not yet on branching (Figure 3F). Growth of basal dendrites of L2/3 pyramidal cells was not affected by prolonged silencing. At DIV 20, the reduced apical dendritic complexity was detectable in the Sholl analysis. The number of total intersections was lower, and the curve of CNO-treated L2/3 pyramidal cells remained below the curve of the control cells between 60 and 250 μm distance from the soma (Figure 3G). Maturation of L5/6 pyramidal cells was neither altered at DIV 10, nor at DIV 20, nor after the recovery. L5/6 cells thus represented a batch-internal control cell population.

Initially, CNO has been considered inert (Armbruster et al., 2007). Yet, CNO becomes metabolized to clozapine and related cell-permeable metabolites, and this way can have a variety of effects depending on the dosage (Löffler et al., 2012; MacLaren et al., 2016; Manvich et al., 2018). Higher concentrations are often used with peripheral application *in vivo*, because the drug has to pass the blood-brain-barrier. A recent study has shown that 10 μM CNO is sufficient to inhibit the binding of natural ligands to GPCRs (Gomez et al., 2017). Our experiments involved barrier-free slice cultures, and we used only 3 μM CNO which has been shown to be sufficient to activate the DREADDs in biophysical assessments with dissociated cells. Further, a medium change every other day counteracted against drug/metabolite accumulation. Application of CNO in the absence of DREADDs has no effect on calcium oscillations (Jendryka et al., 2019) or morphology (MacLaren et al., 2016). Therefore, to ensure that effects on apical differentiation were based on activation of hM4Di and not a result solely of the application of CNO, GFP-only transfected neurons were treated with 3 μM CNO from DIV 5–10. L2/3 pyramidal apical dendrites were not different from controls suggesting that CNO in the absence of hM4Di has no detectable side effects (Supplementary Figure 1). In summary, the observations provided evidence that G protein driven inhibition can have a role in apical dendritic maturation of L2/3 pyramidal cells.

### Silencing *via* HM4Di Impairs Pyramidal Cell Axon Development

**FIGURE 4:**
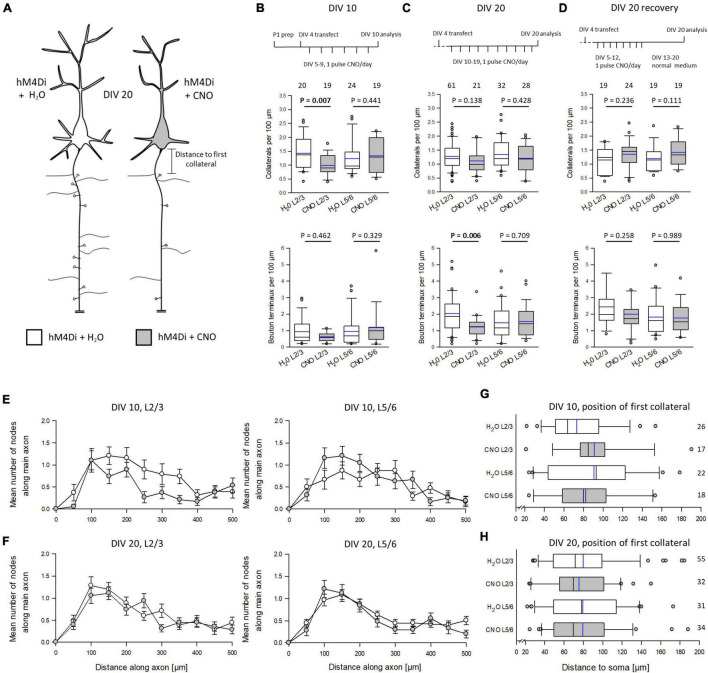
Silencing *via* hM4Di impairs pyramidal cell axon development. **(A)** Prolonged activation of hM4Di was hypothesized to impair axonal differentiation. **(B)** Stimulation protocol, the effect on collateralization, and the effect on the density of bouton terminaux at DIV 10; 3 batches. **(C)** Stimulation protocol, the effect on collateralization, and the effect on the density of bouton terminaux at DIV 20; 4 batches. **(D)** Stimulation protocol, the effect on collateralization, and the effect on the density of bouton terminaux at DIV 20 recovery; 3 batches. **(E)** Distribution of collaterals along the first 500 μm of the main axon of L2/3 and L5/6 pyramidal cells at DIV 10. **(F)** Distribution of collaterals along the first 500 μm of the main axon of L2/3 and L5/6 pyramidal cells at DIV 20. **(G)** Distance to the soma of the first collateral arising from the main axon of L2/3 and L5/6 pyramidal cells at DIV 10. **(H)** Distance to the soma of the first collateral arising from the main axon of L2/3 and L5/6 pyramidal cells at DIV 20. Numbers of analyzed neurons in panels **(B–F)** are given above the boxplots in panels **(B–D)**, and right of the box plots in panels **(G,H)**. Black lines in the box plots represent the median and blue lines indicate the mean. Mann-Whitney rank sum test H_2_O control vs. CNO, *P*-values are reported.

As described above, G_i/o_ signaling has been associated with axonal growth in various neural models. However, we could not detect HA-hM4Di in the axons. Yet, neuronal silencing *per se* may have altered the axonal pattern. We expected hypomorphic axons (Figure 4A). Pyramidal axons are difficult to reconstruct in total because collaterals are much thinner and equipped with delicate boutons when compared to the main axon. Over the distance, axons were not always completely EGFP-labeled. As a proxy, we reconstructed the first ∼500 μm of the main axon of the pyramidal cells (Supplementary Figures 2A–J). In rodent cortex, axons can emerge from the soma as well as from a basal dendrite (Wahle et al., 2022). We determined the number of collaterals and bouton terminaux which are *bona fide* presynapses. Early prolonged silencing significantly reduced the number of collaterals arising from the main axon of L2/3 pyramidal cells. The number of bouton terminaux was not changed at DIV 10, however, the number was extremely low in the DIV 5–10 time window (Figure 4B). In contrast, prolonged silencing of cells at DIV 10–20 was no longer able to reduce the number of collaterals. However, the number of bouton terminaux was now affected because their density increased about 4-fold in L2/3 control cells, but not in CNO-treated L2/3 cells (Figure 4C). This suggested that already formed collaterals are no longer sensitive to G protein-mediated inhibition. However, the formation of presynapses was delayed. Similar to apical dendrites, the collateral density was recoverable until DIV 20, and the bouton terminaux developed similar to those of control cells (Figure 4D).

At DIV 10, the Sholl-type analysis of the collateral distribution revealed a reduction of collateral density along more distal parts of the main axon of L2/3, but not L5/6 pyramidal cells (Figure 4E). The collateral distribution was no longer different from that of L2/3 and L5/6 control cells at DIV 20 (Figure 4F). We also tested if the distance between axon origin from soma or from a basal dendrite differed in CNO-treated neurons. We counted all collaterals arising beyond 20 μm, which corresponds to the axon initial segment where pyramidal cell axons extremely rarely branch, to maximally 200 μm. The distance to the first collateral varied considerably from cell to cell, but it did not vary with treatment at DIV 10 (Figure 4G) or at DIV 20 (Figure 4H). Together, this suggested a role of neuronal silencing on the formation of axon collaterals and presynapses of L2/3 pyramidal cells.

### Silencing *via* HM4Di Subtly Impairs Interneuron Development

**FIGURE 5:**
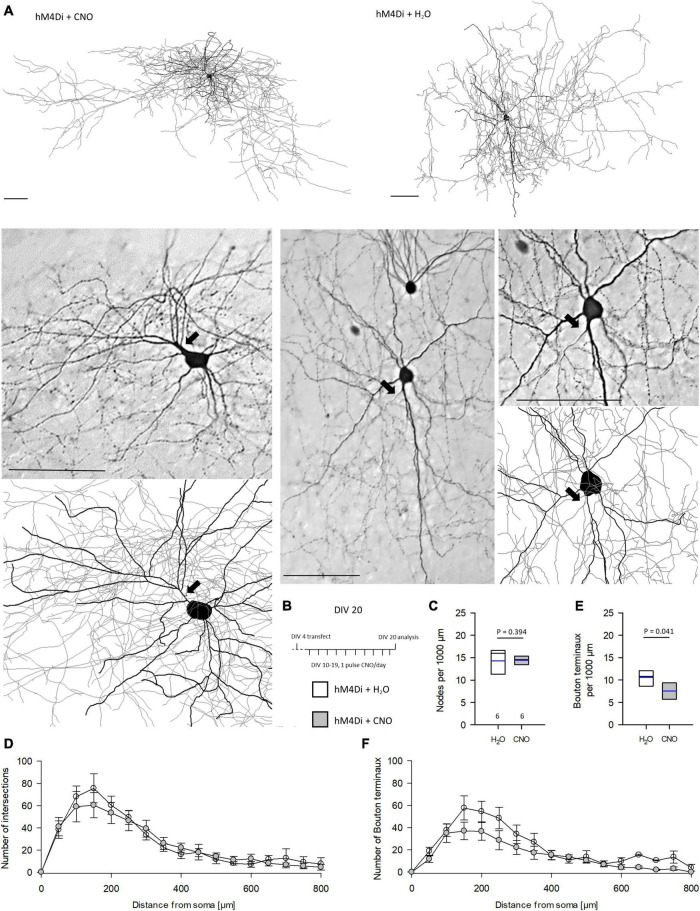
Prolonged reduction of activity *via* hM4Di reduces the number of bouton terminaux of basket cells axons. **(A)** Two representative cells, one each of CNO and H_2_O treatment, are shown as a reconstruction overview (thick lines, dendrites; thin lines, axons), and as photomicrograph and detail of the reconstruction. Axons are marked by arrows. **(B)** Stimulation protocol. **(C)** Number of nodes per 1,000 μm. **(D)** Sholl analysis and total intersections of reconstructed basket cells. **(E)** Number of bouton terminaux per 1,000 μm. **(F)** Sholl analysis of bouton terminaux; note that the density remained below that of control cells over the entire distance. Numbers of analyzed neurons are given below the box plots in panel **(C)**. Black lines in the box plots represent the median and blue lines indicate the mean. Mann–Whitney rank sum test H_2_O vs. CNO, *P*-values are reported. Scale bars: 100 μm.

We reconstructed interneurons at DIV 10, DIV 20, and DIV 20 recovery. Dendritic length and branching was not affected by CNO treatment (Supplementary Table 2). The same was found when analyzing the interneuron subsets. Basket cells with terminal segments contacting somata and cells with vertical interlaminar axons of bitufted, arcade and Martinotti cell morphology were equally well developed compared to control cells (Supplementary Figure 3). Next, we analyzed completely EGFP-stained axons of basket neurons (Figure 5A) in cultures treated with CNO or H_2_O from DIV 10–20 (Figure 5B). The time corresponds to the postnatal period in visual cortex when basket cells form dense local arborizations. Axograms of the two neurons shown in Figure 5A are presented in Supplementary Figure 4A which also gives the raw values of the analyzed basket cell axons (Supplementary Figure 4B). The number of branch points (nodes) per 1,000 μm was the same (Figure 5C) as was the number of intersections in the Sholl analysis (Figure 5D). Interestingly, the number of bouton terminaux per 1,000 μm was reduced by CNO treatment (Figure 5E). This was in line with the reduction of bouton terminaux along the initial portion of L2/3 pyramidal cell axons at DIV 20. The reduction seemed to occur along the entire path since the curve of the CNO-treated basket cell axons remained below the control (Figure 5F).

Further, basket cell axonal boutons were addressed. Axosomatic bouton size is a regulated feature. Typically, these boutons are irregular in size ranging from 0.3 to >1.5 μm^2^. Pyramidal cell axons are not static throughout lifetime, and bouton dynamics, e.g., addition or loss as well as growth or shrinkage, increases with age (Grillo et al., 2013). For instance, in monkey cortex axosomatic boutons grow with age (Soghomonian et al., 2010). In rodent, axosomatic boutons have been shown to remain smaller at DIV 10–20 by inflammation-induced hyperexcitability (Engelhardt et al., 2018). However, silencing of hM4Di-expressing basket neurons did not impair development of bouton size (Supplementary Figures 5A–C). Together, this suggested that numerical development of presynaptic boutons tends to be impaired, while bouton size development seems not affected.

## Discussion

Designer receptors exclusively activated by designer drugs are extensively used in neurophysiological and behavioral studies. Yet, a detailed description of cellular DREADD expression is less often provided. Our stainings of hM4Di in pyramidal cells revealed a heterogeneous distribution in the somatodendritic compartment with enrichment in apical dendrites and less prominent in basal dendrites and axons. Somatic labeling was mainly cytosolic suggesting either nascent HA-tagged hM4Di protein or protein that got stuck at the level of the ER as is often seen with overexpression. The dendritic and axonal localization confirms earlier reports (Armbruster et al., 2007; Stachniak et al., 2014). In hippocampal pyramidal cells, hM4Di signaling leads to a hyperpolarization (Armbruster et al., 2007). GIRK channels are located in soma and apical dendrites of pyramidal cells (Takigawa and Alzheimer, 1999; López-Bendito et al., 2002; Chen and Johnston, 2005) and thus locally close to hM4Di receptors. Accordingly, calcium imaging in hM4Di-expressing cortical neurons revealed a reduction of amplitude with CNO application in soma, dendrites, and also a flattening of calcium events in the proximal axon. The effects were outlasting the acute CNO application. However, it is unlikely to assume that our hMD4i-transfected neurons had remained completely silent during the 5–10 days exposure periods. Our sparse transfections yielded rather few hM4Di-expressing neurons per cultures, which are embedded in a network of thousands of spontaneously active wildtype neural cells. Silencing of a handful of neurons is therefore unlikely to inhibit the entire network. By contrast, transfection of GluK2 into a few pyramidal cells enhances dendritic growth not only of the transfectants, but also of their wildtype neighbors because a few highly active transfectants are capable to increase the activity of the entire network (Jack et al., 2018).

The hM4Di-mediated silencing was sufficient to delay apical dendritic development, axonal collateralization, and development of bouton terminaux of excitatory neurons, and the latter parameter tended to be reduced in basket cell axons. Developing interneurons express G_i/o_-coupled receptors (Gonchar et al., 2001; López-Bendito et al., 2002; Bodor et al., 2005); however, they did not respond to CNO with a dendritic growth delay. For pyramidal cells, the reduction of the calcium events may have caused the growth delay. An interference with calcium homeostasis has been shown to impair dendritic development (Gasterstädt et al., 2020). Besides, signaling cascades may be altered. For instance, depending on the presence of β-arrestin, a GPCR-β-arrestin complex can form which can promote ERK phosphorylation (Smith et al., 2021) with cytosolic retention and impairment of nuclear ERK signaling (Tohgo et al., 2002). Membrane to nucleus transcriptional signaling, however, is important for dendritic growth. Further, excitatory as well as inhibitory GPCR signaling can lead to transactivation and internalization of receptor tyrosine kinases including receptors implicated for dendritic growth (Di Liberto et al., 2019; Kilpatrick and Hill, 2021). For instance, in cerebellar neurons activating GABA_B_ receptors enhances survival *via* transactivation of insulin growth factor-1 receptor signaling (Tu et al., 2010).

Pyramidal cells respond to hM4Di-mediated silencing in a compartment- and layer-specific manner. The apical growth delay can be detected only in L2/3 pyramidal dendrites. Especially in apical dendrites with their lower surface-to-volume ratio, GABA_B_/GIRK channel-mediated currents reduce the influence of depolarizing apical dendritic inputs on axonal action potential output (Schulz et al., 2021). The unimpaired basal dendritic complexity could either be due to low expression of hM4Di receptors or of GIRK channels in basal dendrites, or could be attributed to different growth mechanisms. Layer- and compartment-specific effects on dendritic differentiation have been reported for ionotropic receptors. For instance, L2/3 pyramidal cells respond with apical growth to overexpression of AMPA and kainate receptor subunits while L5/6 cells do not (Hamad et al., 2011; Jack et al., 2018). In contrast, basal dendritic growth requires active NMDA receptors (Gonda et al., 2020). Layer-specific effects are also seen for gene expression in that visual experience can drive transcription after eye opening in visual cortex L2/3 neurons, while those of L5/6 become instructed prior to eye opening (Cheng et al., 2022). This suggested that in particular for L2/3 pyramidal cells any subtle increase of excitability will lead to accelerated dendritic growth, while a subtly higher level of inhibition as with hM4Di activation is sufficient to delay dendritic maturation.

With regard to metabotropic signaling GABA_B_ receptors are well expressed already prenatally with enrichment in pyramidal cell apical dendrites and in subsets of interneurons (López-Bendito et al., 2002). A knockdown of the GABA_B_2 receptor impairs perinatal migration by shortening the leading processes of pyramidal neurons *en route* to L2/3 (for review Gaiarsa and Porcher, 2013) which may delay development of apical dendrites. Also, premature expression of the chloride transporter KCC2 substantially reduces dendritic maturation of L2/3 pyramidal cells (Cancedda et al., 2007). Therefore, premature hyperpolarizing actions of GABA disrupt pyramidal development.

The effects reported here look rather moderate. Technically, we reconstructed completely and mostly intensely EGFP-stained neurons, and although coexpression rates are high with biolistic transfection (Wirth et al., 2003; Hamad et al., 2011) the hM4Di expression levels might well have varied from cell to cell. Biologically, it has been reported that many cortical neurons are naturally fairly silent. In mouse somatosensory cortex 70% of the information flow is managed by a so-called rich-club of ∼20% hub neurons which have a lion’s share of input and output connections, while many neurons are rarely or never recruited (Nigam et al., 2016). Indeed, we saw hM4Di-transfected neurons, which were not spontaneously active during the few minutes of recording. However, we can not conclude that these neurons had been inactive throughout the culturing period. It is not possible to determine the amount of active and silent neurons in every single culture and select for reconstruction only those cells which had been active before CNO treatment. Moreover, in particular L2/3 pyramidal cells are reported to be under strong GABA-ergic inhibition (Petersen and Crochet, 2013). All this has worked against us. However, the batch-internal proportion of naturally silent cells should affect the control and the CNO-treated slice cultures equally. Further, although many neurons in our culture would not have fired at high rates, they may have well received membrane depolarizations sufficient for sustaining dendritic growth. These reasons may explain why the effect of additional Gi-evoked inhibition has been subtle.

Nevertheless, even a moderate impairment of dendritic morphology may have a substantial functional impact. A recent report shows that large L5 pyramidal neurons of primary and secondary cortical areas differ in apical length, and the shorter the dendrite the more reduced is the cell’s dendritic excitability and propensity of burst firing (Galloni et al., 2020). A precise regulation of the dendritic architecture is a prerequisite of neural computations, especially with regard to the newly proposed model on deep learning in even single dendritic branches (Hodassman et al., 2022).

The present study found a higher sensitivity to hM4Di-mediated inhibition of L2/3 pyramidal cells between DIV 10–20. Effects evoked by CNO treatment between DIV 5–10 were milder in that apical elongation, but branching was not impaired. Effect strength may depend on the efficiency of the coupling between G_i/o_ receptor signaling and GIRK channels. Coupling strength has been reported to be low early postnatally for GABA_B_ receptors (Bony et al., 2013), although, functional GABA_B_ receptor action occurs in many isolated cortical pyramidal neurons already by postnatal day 3 (Sickmann and Alzheimer, 2002). The expression of the endogenous m4 receptor mRNA increases until postnatal day 10 in cortical layers (Rossner et al., 1993) being strongest at postnatal day 5 (Hohmann et al., 1995). The transfection at DIV 4 presumably resulted in hM4Di expression parallel (or even accelerated) with the endogenous m4 receptor expression. The substantial somatodendritic HA-hM4Di labeling of pyramidal cells in cultures supported this assumption. Coupling of muscarinic cholinergic receptors to GIRK channels may progress equally slowly postnatally, possibly because cholinergic afferents slowly start to invade the cortical layers during the first postnatal week in rat (Mechawar and Descarries, 2001).

Early G protein mediated inhibition of L2/3 pyramidal cells resulted in a delayed development of axonal collateralization and bouton terminaux which are *bona fide* presynapses and which are used here as a proxy of presynapse development. These effects were time-dependent: after DIV 10 the collateralization could no longer be altered, yet presynapses became malleable. Similar to the dendritic impairment, the collateralization deficit was reversible when activity was allowed to resume. GPCRs are known to influence axonal growth. For example, GABA_B_ receptor activation reduces neurite growth in olfactory receptor neurons (Priest and Puche, 2004) and negatively regulates neurite growth of mouse spinal neurons (Bird and Owen, 1998). Acetylcholine has been shown to inhibit the growth of thalamic axons by reducing growth cone motility. This effect was most sensitive to oxotremorine sesquifumarate (Rüdiger and Bolz, 2008), an antagonist with preference for G_i/o_-coupled M2 receptors. Our results suggest that the reduction of somatodendritic excitability of hM4Di-expressing neurons had also reduced the axonal output and had delayed collateral and presynapse development. In line with this assumption, blocking action potential activity with tetrodotoxin disrupts the layer-specific formation of collaterals of L2/3 and L6, but not L5 pyramidal neurons in cultures from early postnatal ferret cortex (Dantzker and Callaway, 1998; Butler et al., 2001).

## Conclusion

We show that repetitive G_i/o_-mediated silencing can delay dendritic and axonal development of cortical neurons. Effects are reversible and recover when activity resumes. Results suggest that G_i/o_ signaling during postnatal development may counterbalance the strong growth-promoting influences of activity-dependent neurotrophins, extracellular cues and depolarization by dampening the calcium signaling.

## Data Availability Statement

The original contributions presented in the study are included in the article/Supplementary Material, further inquiries can be directed to the corresponding author.

## Ethics Statement

The animal study was reviewed and approved by the Ruhr University Bochum Animal Research Board and the Federal State of North Rhine-Westphalia.

## Author Contributions

PW conceived the experiments. IG, MS, LC, JK, L-MR, BM, AJ, and PW performed the experiments and data management. IG, SH, AJ, and PW interpreted results. IG and PW wrote the manuscript. All authors contributed to the article and approved the submitted version.

## Conflict of Interest

The authors declare that the research was conducted in the absence of any commercial or financial relationships that could be construed as a potential conflict of interest.

## Publisher’s Note

All claims expressed in this article are solely those of the authors and do not necessarily represent those of their affiliated organizations, or those of the publisher, the editors and the reviewers. Any product that may be evaluated in this article, or claim that may be made by its manufacturer, is not guaranteed or endorsed by the publisher.
