# Supplementary material for: Chemogenetic Silencing of Differentiating Cortical Neurons Impairs Dendritic and Axonal Growth
Source: Front Cell Neurosci. 2022 Jul 15;16:941620. doi: 10.3389/fncel.2022.941620 (PMC9336219; doi:10.3389/fncel.2022.941620)
Supplement: Supplementary file 1 [file Data_Sheet_1.PDF]

Supplementary Material

Supplementary Table 1. Measures of apical and basal dendrites of hM4Di transfected pyramidal neurons of L2/3 and L5/6 stimulated with CNO versus batch-internal hM4Di expressing neurons stimulated with H_2O as control at DIV 10, DIV 20 and DIV 20 recovery. Given is the mean ± s.e.m., and n, the number of cells analyzed. Statistics: Mann-Whitney rank sum test H_2O control versus CNO, P-values are shown in italic. ADL, apical dendritic length (µm); BDL, mean (per cell) basal dendritic length (µm); segments, number of dendritic segments.

Supplementary Table 1. HM4Di transfected pyramidal cells stimulated with CNO at DIV10, DIV20 and DIV20 recovery

	Pyramidal cells in L2/3		Pyramidal cells in layers L5/6	
Age, Condition<br>(Number of batches)	ADL (n)<br>Segments	BDL<br>Segments	ADL (n)<br>Segments	BDL<br>Segments
**DIV 10**<br>Control (7)	1418 ± 62 (51)<br>31.1 ± 1.7	285 ± 19<br>7.3 ± 0.4	1131 ± 74 (41)<br>24.4 ± 2.1	277 ± 21<br>6.6 ± 0.5
CNO	1282 ± 59 (78)<br>28.9 ± 1.4	318 ± 24<br>7.6 ± 0.4	1161 ± 89 (36)<br>25.5 ± 2.4	228 ± 15<br>5.9 ± 0.3
Mann-Whitney test	***0.043***<br>*0.239*	*0.541*<br>*0.666*	*0.996*<br>*0.782*	*0.163*<br>*0.431*
**DIV 20**<br>Control (8)	1656 ± 66 (118)<br>30.1 ± 1.0	320 ± 13<br>6.7 ± 0.3	1435 ± 66 (94)<br>25.3 ± 1.1	328 ± 15<br>6.5 ± 0.3
CNO	1400 ± 76 (67)<br>25.1 ± 1.3	338 ± 17<br>6.9 ± 0.3	1379 ± 83 (74)<br>23.9 ± 1.4	331 ± 18<br>6.6 ± 0.3
Mann-Whitney test	***0.011***<br>***0.001***	*0.365*<br>*0.430*	*0.377*<br>*0.166*	*0.835*<br>*0.866*
**DIV 20 recovery**<br>Control (4)	1638 ± 82 (42)<br>29.9 ± 1.3	396 ± 21<br>8.8 ± 0.5	1823 ± 118 (32)<br>30.4 ± 2.1	403 ± 23<br>7.8 ± 0.5
CNO	1770 ± 95 (43)<br>32.1 ± 2.4	413 ± 23<br>9.3 ± 0.5	1833 ± 142 (32)<br>30.8 ± 2.4	395 ± 24<br>8.3 ± 0.6
Mann-Whitney test	*0.268*<br>*0.715*	*0.785*<br>*0.650*	*0.984*<br>*0.989*	*0.699*<br>*0.716*

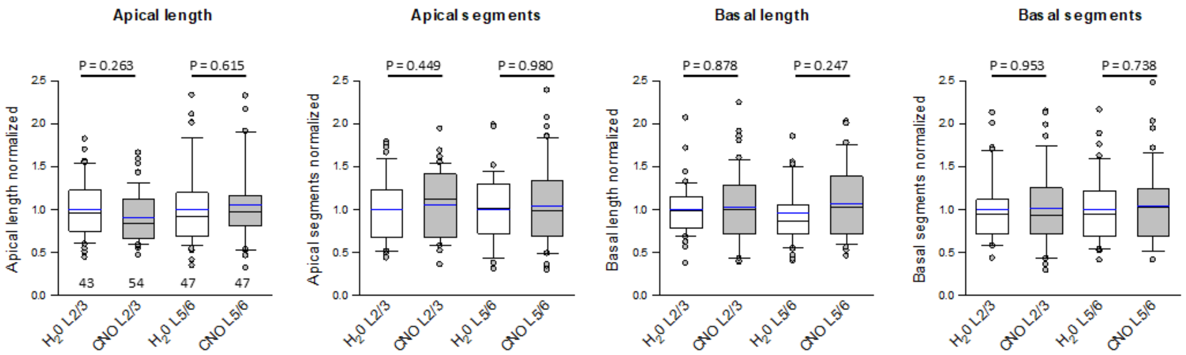

Supplementary Figure 1. Measures of apical and basal dendrites of EGFP-only transfected pyramidal neurons of L2/3 and L5/6 treated with CNO versus batch-internal EGFP expressing neurons treated with H_2O as control at DIV 20. Given is the mean ± s.e.m., and n, the number of cells analyzed; 3 independent preparations. Black lines in the box plots represent the median and blue lines indicate the mean. Statistics: Mann-Whitney rank sum test H_2O control versus CNO, P-values are shown in italic. ADL, apical dendritic length (µm); BDL, mean (per cell) basal dendritic length (µm); segments, number of dendritic segments.

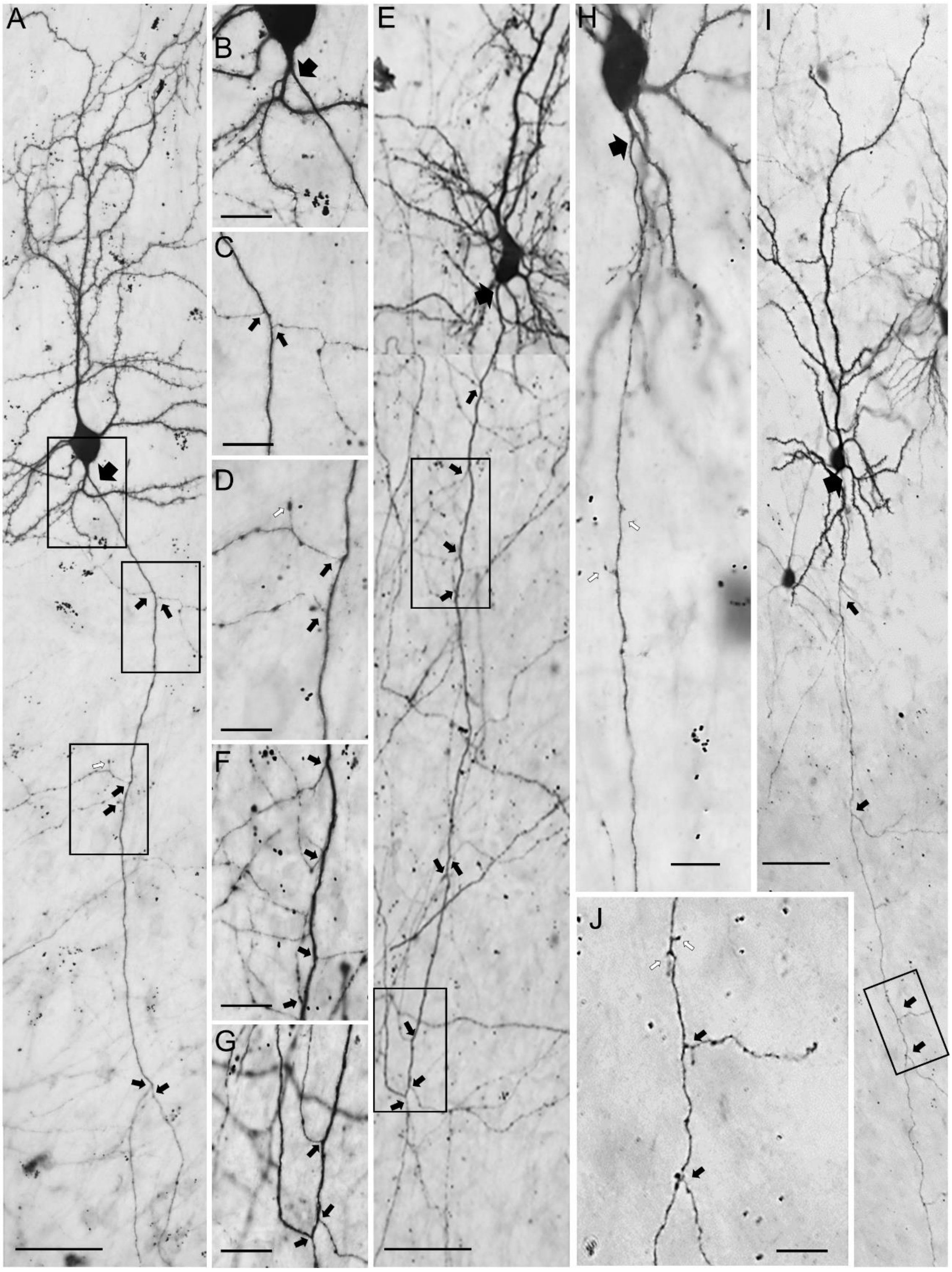

Supplementary Figure 2. Axon morphology. **(A), (E) (H)** and (**I**) show photomontages of L2/3 pyramidal neurons with their main axon descending towards the white matter. (**A, E**) DIV 10, H_2O, (**H**) DIV 10 CNO, (**I**) DIV 20 H_2O. Large arrows indicate the origin of the axon from a basal dendrite (**A**, **H**) or the soma (**E, I**). Small filled arrows indicate collaterals arising from the main axon. Small open arrows indicate bouton terminaux. **(B), (C)** and **(D)** are higher magnifications of the insets in **(A)**. **(F)** and **(G)** represent the insets in **(E)** and show collaterals. (**J**) is the inset in (**I**) showing collaterals and bouton terminaux. Scale bars: 50 µm in A, E, I, and 15 µm in B, C, D, F, G, H, J.

Supplementary Table 2. Measures of dendrites of hM4Di transfected multipolar interneurons stimulated with CNO versus batch-internal hM4Di expressing neurons stimulated with H_2O as control at DIV 10, DIV 20 and DIV 20 recovery. Given is the mean ± s.e.m., and n, the number of cells analyzed. Statistics: Mann-Whitney rank sum test H_2O control versus CNO, P values are shown in italic. MDL, mean (per cell) dendritic length (µm); MDS, mean (per cell) basal dendritic segments; PD, number of primary dendrites.

Supplementary Table 2. HM4Di transfected multipolar interneurons stimulated with CNO at DIV10, DIV20 and DIV20 recovery

Condition<br>(Number of batches)	MDL (n)<br>MDS	PD	MDL (n)<br>MDS	PD	MDL (n)<br>MDS	PD
	**DIV 10**		**DIV 20**		**DIV 20 recovery**	
Control<br>(7, 8, 4 batches, resp.)	444 ± 41 (25)<br>7.6 ± 0.8	4.1 ± 0.3	537 ± 31 (88)<br>7.8 ± 0.4	5.1 ± 0.2	561 ± 36 (68)<br>7.4 ± 0.5	5.5 ± 0.2
CNO	399 ± 20 (32)<br>7.1 ± 0.5	4.2 ± 0.2	481 ± 21 (103)<br>6.9 ± 0.3	4.5 ± 0.1	495 ± 28 (46)<br>6.9 ± 0.5	5.1 ± 0.2
Mann-Whitney test	*0.694*<br>*0.764*	*0.987*	*0.256*<br>*0.421*	*0.412*	*0.410*<br>*0.187*	*0.024*

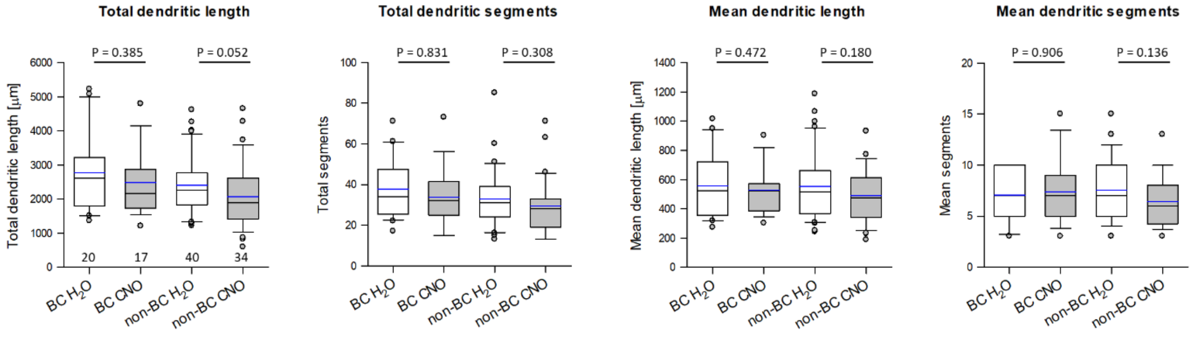

Supplementary Figure 3. Measures of dendrites of hM4Di transfected basket cells and vertically projecting non-basket cells stimulated with CNO versus batch-internal hM4Di expressing neurons stimulated with H_2O as control at DIV 10 and DIV 20. Numbers of analyzed neurons are given below the box plots. Mann-Whitney rank sum test H_2O control versus CNO, P-values are reported.

A

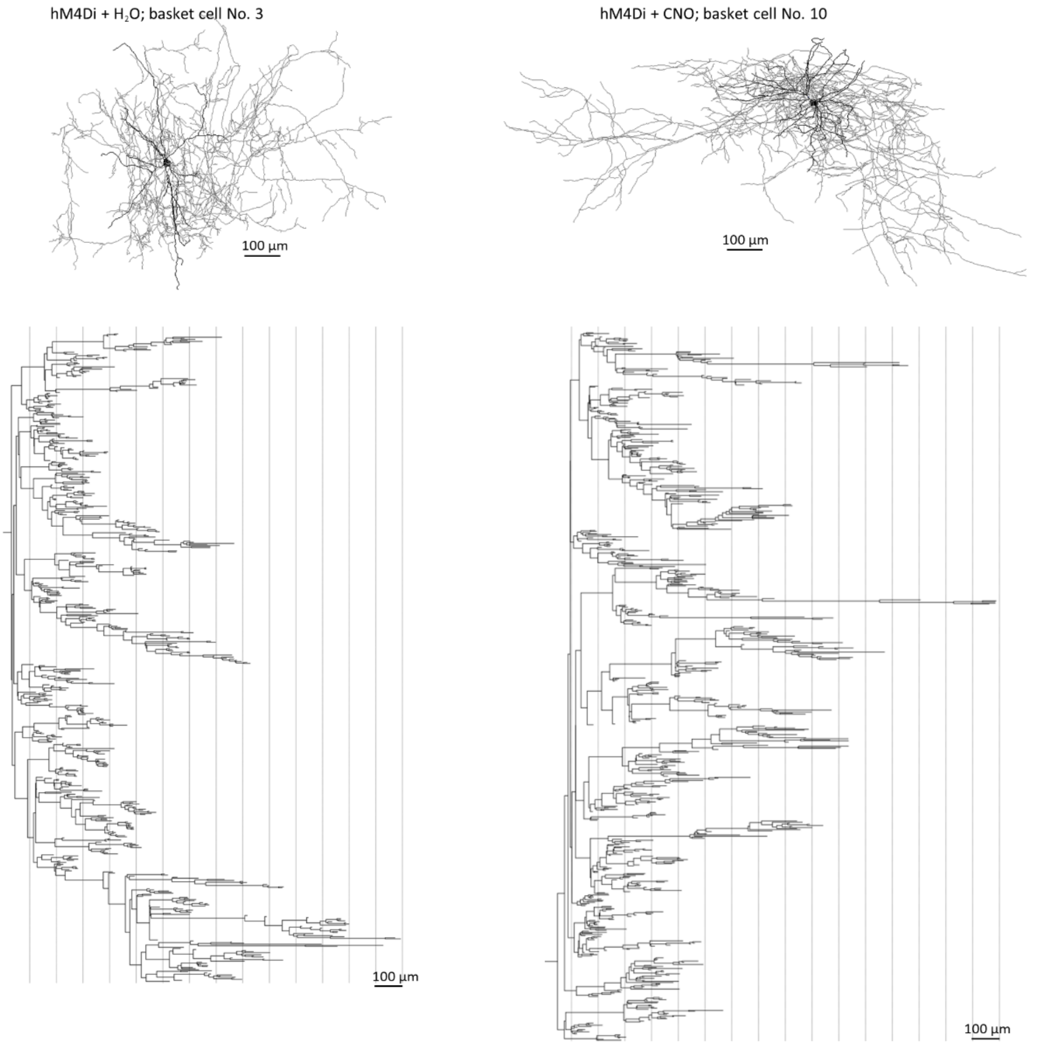

B

HM4Di transfected basket cells stimulated with CNO at DIV20				
Condition	Cell number	Total axon length [µm]	Total nodes	Total bouton terminaux
Control	1	34,787	283	405
	2	12,989	201	103
	3	37,075	609	497
	4	36,736	453	406
	5	35,828	605	317
	6	31,855	527	338
CNO	7	7,030	103	43
	8	32,387	497	242
	9	38,495	553	420
	10	50,657	661	229
	11	35,237	485	313
	12	26,766	411	203

Supplementary Figure 4. Basket cell axon data. (**A**) The two representative neurons as reconstruction and as axograms generated with the Neurolucida program. (**B**) Total length, nodes, and bouton terminaux of the axons analyzed.

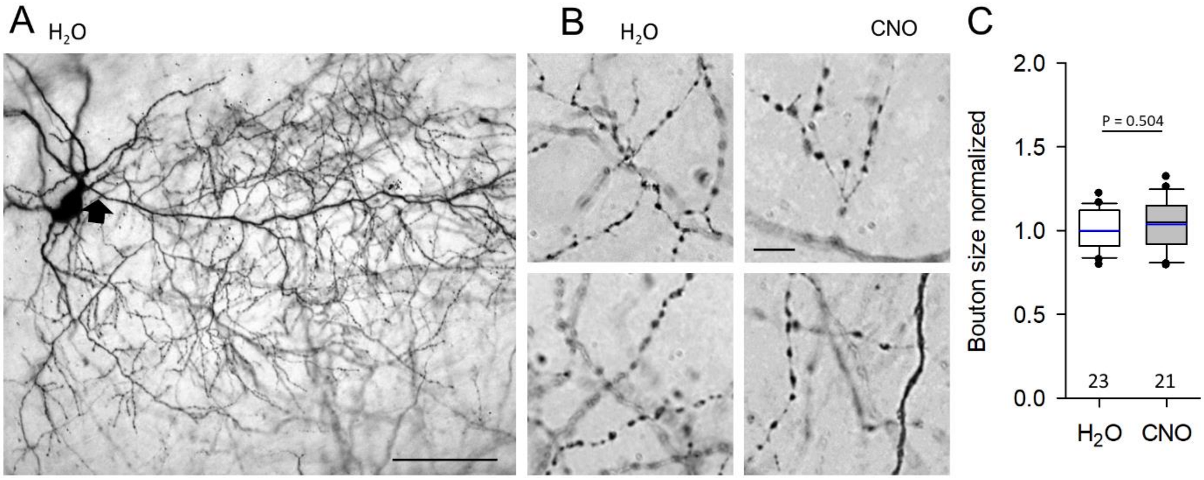

Supplementary Figure 5. Bouton size of hM4Di transfected basket cells was not altered after CNO stimulation at DIV 20. **(A)** Representative photomicrograph of one of the analyzed basket cell axons. The origin is marked by a large arrow. **(B)** Representative photomicrographs of boutons of hM4Di transfected cells stimulated with H_2O (left) and CNO (right). **(C)** Bouton size normalized to the average of the batch-internal control, 3 independent preparations. Scale: 100 µm in A and 5 µm in B. Numbers of analyzed neurons are given below the boxplots. Black lines in the box plots represent the median and blue lines indicate the mean. Per axon, a minimum of 30 to a maximum of 149 boutons were measured on photomicrographs taken within every plexus arbitrarily from at least 8-15 axonal segments (2.487 boutons in total, 56 boutons on average per cell). Bouton measures were condensed to one value per cell. Statistics: Mann-Whitney U rank sum test H_2O control versus CNO, P-value is reported.
